# Cancer Genomics: Large-Scale Projects Translate into Therapeutic Advances

**DOI:** 10.1371/journal.pmed.1002209

**Published:** 2016-12-27

**Authors:** Elaine R. Mardis, Marc Ladanyi

**Affiliations:** 1 The McDonnell Genome Institute, Washington University, St. Louis, Missouri, United States of America; 2 Department of Pathology and Human Oncology & Pathogenesis Program, Memorial Sloan Kettering Cancer Center, New York, New York, United States of America

## Abstract

Elaine Mardis and Marc Ladanyi discuss how large-scale genomics has driven advances in cancer translational medicine, with a focus on publications in this month's Special Issue.

Oncology has, arguably, led the development of precision medicine in recent decades, with genomic and molecular advances informing the development of targeted therapies that have revolutionized treatment of certain individual cancer types. The cancer genomics arena is particularly exciting at the present time for two reasons: many groups across the world have been working hard in recent years to characterize tumor genomes and to report their findings from a tissue site–specific and a pan-cancer perspective. This huge body of genomic information generated by The Cancer Genome Atlas (TCGA), The International Cancer Genome Consortium (ICGC), and The Pediatric Cancer Genome Project has contributed significantly to our understanding of individual cancer types. There has, importantly, also been a huge expansion of technical advances, including devices to support next-generation sequencing, high-sensitivity digital droplet PCR, methods for analysis of cell-free DNA, new error correction approaches for ultra-deep sequencing, and novel computational analysis tools. Together, these efforts have provided a golden opportunity for researchers to further mine the available data and begin to develop diagnostic and clinical applications to benefit patients.

There have been questions regarding whether large-scale cancer discovery genomics projects were the best use of limited research funds—in terms of the opportunity cost of funding large-scale rather than multiple investigator–initiated research projects. Because we would argue strongly for the value of these large-scale projects, it is particularly gratifying that many papers in this special issue of *PLOS Medicine* strongly testify to the realization of the anticipated downstream impacts of these coordinated sequencing efforts by using large-scale data sets as a starting point for data mining, hypothesis generation, or as comparator data. Their value is further reflected in the intellectual communities that were created as a result of publicly and privately funded large-scale research and the new lines of research inquiry that originated from their novel findings. Finally, the improved technical standards throughout the cancer genomics field, both in terms of sequencing data quality and bioinformatics, ensure that the community will continue to benefit substantially in scientific advances from these landmark projects.

As part of this special issue, research articles and perspectives on cancer genomics are appearing in *PLOS Medicine* throughout December 2016. These papers focus on a wide range of different cancers and experimental approaches and yield findings relevant both to future scientific research and potential clinical advances—here, we will highlight just a few of these excellent studies. Two research papers report on the profiling of the mutational landscape of widely metastatic breast cancer, from Charles Perou and colleagues at UNC Chapel Hill, United States [1], and from Sherene Loi and colleagues at the Peter MacCallum Cancer Centre in Melbourne, Australia [2]. These studies are based on the sampling of multiple sites of metastatic disease, something that usually can only be performed at the time of autopsy. To obtain high quality tissue samples for sequencing, these autopsies must be performed relatively soon after death. These studies are therefore logistically demanding to carry out because patients must be approached for the rapid autopsy consent, and while participants often die at home or in hospice, their bodies must be transported in a specific timeframe to an autopsy suite where a pathologist must be on standby to perform the associated procedure—all before the samples can be banked for subsequent study. Perou and colleagues studied primary and metastatic tumors from two women with triple-negative breast cancer, aiming to understand the genomic events accompanying progression and metastasis of this hard-to-treat form of cancer. In Loi and colleagues’ study, the authors profiled metastatic tumors from four breast cancer patients obtained at autopsy. The existence of hereterogeneity between both the primary and multiple metastatic tumors is revealed in all cases and indicates that treatment drove subclonal diversification and, in some cases, the parallel evolution of resistance in metastatic tumors. The genomics of metastatic breast cancers have until recently remained largely understudied [3,4], and these papers set the stage for larger-scale projects to come. Initial studies on intratumoral heterogeneity caused some anxiety in terms of how to make sense of this heterogeneity and of its implications for targeted therapies [5]. The above contributions illustrate how these emerging data on the clonal evolution or tumor architecture of widely metastatic cancer should help in selecting the most appropriate molecular targets for therapy in the setting of tumor heterogeneity.

In a study from James Brenton and colleagues from the Cancer Research UK Institute in Cambridge, United Kingdom, the authors analyzed circulating tumor DNA (ctDNA), specifically circulating DNA bearing TP53 mutations in women with high-grade serous ovarian cancer (HGSOC), and compared its sensitivity to conventional imaging and CA-125 level monitoring of relapse [6]. TP53 mutations constitute the optimal genetic marker in this cancer as they are present in nearly 100% of HGSOC tumors, as established by TCGA and other genomic studies. In addition, from an experimental design point, this is one of the first published studies assessing ctDNA in comparison to a commonly clinically tracked biomarker as well as imaging modalities. There is a high level of activity in this area of research that should build a case for ctDNA monitoring being done routinely in clinical practice, so this retrospective study is important in laying the foundations to change the standard of care.

In another nod to studies emerging from TCGA, Anindya Dutta and colleagues from the University of Virginia Health Sciences System, US, characterized lncRNAs in low-grade gliomas and glioblastoma multiforme (GBM) from RNAseq data [7]. The authors use the lncRNA profiles to provide prognostic information on these hard-to-treat cancers. The authors mined TCGA RNAseq data for lncRNAs, an analysis that was not part of the TCGA’s remit at the time of its initial GBM analysis. In asking what we can learn from this data mining exercise, the authors provide a hypothesis and a predictive model that will likely fuel other studies. While this study is still at the stage of a discovery analysis, it should bring the community closer to the point of clinically translating their findings for this subset of highly aggressive brain cancers. This kind of study represents one of the rationales for large genomic studies with data deposition in public repositories—here is a group looking at the data in a fresh way that was unanticipated at the time of some of these genomic studies and formulating interesting hypotheses that should spark exploration in new patient samples.

In another research article, Jules Meijerink and colleagues from The Princess Maxima Center for Pediatric Oncology, Utrecht, The Netherlands, have investigated a challenging problem in pediatric oncology—mechanisms involved in steroid resistance that underlie poor outcomes in T cell acute lymphoblastic leukemia (T-ALL) [8]. Using whole-genome and exome sequencing, the authors found mutations affecting the interleukin 7 signaling pathway in patients and then demonstrated that signaling inhibitors could bolster the effects of steroids on primary cells from pediatric T-ALL patients. This study is a strong example of integrating genomics with other techniques in a concerted way to address a difficult clinical problem. Drugs targeting some of the signaling molecules identified by Meijerink and colleagues have already undergone clinical development and received approval for other types of cancer, and hence these important results could soon lead to early-stage clinical evaluation of these agents in children with steroid-resistant T-ALL and perhaps in other forms of ALL.

In summary, the robust response from the cancer genomics community to our call for papers has been quite gratifying. In particular, we both find it hugely satisfying to see so many studies building on the findings of large-scale discovery projects, further emphasizing their importance. Finally, we would like to thank all the authors for submitting their work, the patients and their families who consent to and thereby support these studies through their generous donations of tissues and clinical data, and to our cadre of outstanding peer reviewers. This extraordinary combination has made this special issue so exciting and relevant. *PLOS Medicine* will continue to welcome submissions in the topic area, and we look forward to the scientific and clinical achievements to come.

## Abbreviations

ctDNA: circulating tumor DNA
GBM: glioblastoma multiforme
HGSOC: high-grade serous ovarian cancer
ICGC: International Cancer Genome Consortium
T-ALL: T cell acute lymphoblastic leukemia
TCGA: The Cancer Genome Atlas

## References

[1] Perou C, Hoadley KA, Siegel MB, Kanchi KL, Miller CA, et al Tumor Evolution in Two Patients with Basal-like Breast Cancer: A Retrospective Genomics Study of Multiple Metastases. PLoS Med. 2016;13(12):e1002174 10.1371/journal.pmed.1002174 27923045 PMC5140046

[2] Loi S, Savas P, Teo ZL, Lefevre C, Flensburg C, et al The Subclonal Architecture of Metastatic Breast Cancer: Results from a Prospective Community-Based Rapid Autopsy Program "CASCADE". PLoS Med. 2016;13(12):e1002204 10.1371/journal.pmed.1002204 28027312 PMC5189956

[3] Shah SP, Morin RD, Khattra J, Prentice L, Pugh T, Burleigh A et al Mutational evolution in a lobular breast tumour profiled at single nucleotide resolution. Nature. 2009;461(7265):809–13. 10.1038/nature08489 19812674

[4] Ding L, Ellis MJ, Li S, Larson DE, Chen K, Wallis JW et al Genome remodelling in a basal-like breast cancer metastasis and xenograft. Nature. 2010 4 15;464(7291):999–1005. 10.1038/nature08989 20393555 PMC2872544

[5] Gerlinger M, Rowan AJ, Horswell S, Larkin J, Endesfelder D, Gronroos E et al Intratumor heterogeneity and branched evolution revealed by multiregion sequencing. N Engl J Med. 2012 3 8;366(10):883–92. 10.1056/NEJMoa1113205 22397650 PMC4878653

[6] Brenton J, Parkinson CA, Gale D, Piskorz A, Biggs H, et al Exploratory Analysis of TP53 Mutations in Circulating Tumour DNA as Biomarkers of Treatment Response for Patients with Relapsed High-Grade Serous Ovarian Carcinoma: A Retrospective Analysis. PLoS Med. 2016;13(12):e1002198 10.1371/journal.pmed.1002198 27997533 PMC5172526

[7] Dutta A, Reon BJ, Anaya J, Zhang Y, Mandell J, et al Expression of lncRNAs in Low-Grade Gliomas and Glioblastoma Multiforme: An In Silico Analysis. PLoS Med. 2016;13(12):e1002192 10.1371/journal.pmed.1002192 27923049 PMC5140055

[8] Li Y, Buijs-Gladdines JG, Canté-Barrett K, Stubbs AP, Vroegindeweij EM, et al IL-7 Receptor Mutations and Steroid Resistance in Pediatric T Cell Acute Lymphoblastic Leukemia: A Genome Sequencing Study. PLoS Med. 2016;13(12):e1002200 10.1371/journal.pmed.1002200 27997540 PMC5172551

